# The diagnostic and prognostic utility of risk factors defined by the AHA/ACCF on the evaluation of cardiac disease in liver transplantation candidates

**DOI:** 10.1186/s12872-019-1088-1

**Published:** 2019-05-02

**Authors:** Sarah Alexander, Meron Teshome, Hena Patel, Edie Y. Chan, Rami Doukky

**Affiliations:** 1 Division of Cardiology, Rush University Medical Center, Chicago, IL USA; 2 Division of Cardiology, Cook County Health, 1901 W. Harrison St., Suite # 3620, Chicago, IL 60612 USA; 3 Department of Surgery, Rush University Medical Center, Chicago, IL USA

**Keywords:** Liver, Transplantation, Coronary artery disease, Diagnosis, Prognosis

## Abstract

**Background:**

The diagnostic and prognostic utility of risk factors proposed by the 2012 American Heart Association and American College of Cardiology Foundation (AHA/ACCF) Scientific Statement on the cardiac assessment of asymptomatic liver transplantation candidates have not been validated. We investigated whether the sum of risk factors proposed by the AHA/ACCF can identify liver transplant candidates at increased cardiac risk.

**Methods:**

In a retrospective cohort of consecutive liver transplantation recipients, we calculated, for each subject, the pre-transplantation sum of AHA/ACCF risk factors (age > 60 years, prior cardiovascular disease, hypertension, dyslipidemia, diabetes mellitus, smoking, and left ventricular hypertrophy). The primary outcome was the presence of severe coronary artery disease (CAD), defined as ≥70% stenosis or ≥ 50% left main stenosis on pre-transplantation angiography. The secondary outcomes were the composite of cardiac death or myocardial infarction (MI) and the composite of cardiac death, MI, or coronary revascularization.

**Results:**

Among 220 liver transplant recipients, the sum of AHA/ACCF risk factors had good discriminatory capacity for severe CAD [area under the curve, 0.77; 95% confidence interval (CI), 0.62–0.92; *P* = 0.007]; having ≥3 risk factors provided 75% sensitivity and 77% specificity for severe CAD. During mean post-transplantation follow-up of 48 ± 31 months, having ≥3 risk factors was associated with increased risk of the secondary composite outcomes of cardiac death or MI [hazard ratio, 2.39; *P* = 0.044] and cardiac death, MI, or coronary revascularization [hazard ratio, 2.39; *P* = 0.044].

**Conclusions:**

In patients undergoing cardiac assessment prior to liver transplantation, the sum of risk factors proposed by the AHA/ACCF provides significant diagnostic and prognostic utility. Having ≥3 AHA/ACCF risk factors is a reasonable threshold to prompt non-invasive stress testing in asymptomatic liver transplantation candidates.

## Background

There is increased awareness of the burden of coronary artery disease (CAD) in patients with end-stage liver disease and worsened outcomes in patients with CAD who undergo liver transplantation [1–6]. In the present day, patients with end-stage liver disease are living longer, such that aging contributes to increased CAD risk. Nonalcoholic fatty liver disease is becoming a more common cause of liver cirrhosis and almost half of these patients have metabolic syndrome which leads to increased CAD risk [5, 7]. Although the increased prevalence of CAD and its attendant long-term consequences in the post liver transplantation patient are known, there is no consensus regarding optimal cardiac risk assessment before liver transplantation and each institution uses its own protocol for risk assessment. In 2012, the American Heart Association and the American College of Cardiology Foundation (AHA/ACCF) published a scientific statement regarding cardiac evaluation in kidney and liver transplantation candidates. The statement indicates that “noninvasive stress testing may be considered in liver transplantation candidates with no active cardiac conditions on the basis of the presence of multiple CAD risk factors regardless of functional status.” [8, 9] These risk factors include age > 60 years, prior cardiovascular disease, hypertension, dyslipidemia, diabetes mellitus, smoking, and left ventricular hypertrophy [8]. The burden of these risk factors that should prompt non-invasive stress testing in asymptomatic patients has yet to be determined. The AHA/ACCF committee does not specify the number of risk factors that should be present to justify stress testing in asymptomatic patients, but “considers 3 or more to be reasonable.” [8] Notably, this is a class IIB recommendation (may be reasonable) with level of evidence C (based on expert opinion, usefulness is unknown/unclear/uncertain or not well established). Therefore, there is need for scientific evidence evaluating the use of the AHA/ACCF risk factors in cardiac risk assessment prior to liver transplantation.

In this investigation, we sought to validate the diagnostic and prognostic utility of the sum of AHA/ACCF risk factors, and determine the threshold sum of risk factors which should prompt further evaluation for CAD with non-invasive stress testing.

## Methods

We conducted a retrospective cohort study of consecutive liver transplantation recipients, who had no cardiac symptoms, from July 2006 to October 2013 at Rush University Medical Center.

### Clinical data

Baseline demographics, cardiovascular risk factors, cardiovascular history, and medications taken prior to liver transplantation were tabulated. Electrocardiograms obtained at the time of preoperative cardiac evaluation were reviewed by observers blinded to cardiac history and clinical outcomes. Left ventricular hypertrophy was defined according to the standard electrocardiographic criteria of Sokolow-Lyon, Cornell, R wave in lead 1 > 14 mV, Gubner-Ungerlieder, and R wave in aVL > 11 mV [10]. The sum of AHA/ACCF risk factors (i.e., age greater than 60 years, hypertension, dyslipidemia, diabetes mellitus, prior cardiovascular disease, tobacco abuse, and left ventricular hypertrophy) present prior to liver transplantation was determined for each patient. The left ventricular ejection fraction as determined by pre-transplantation echocardiogram was recorded. The Model End-stage Liver Disease (MELD) score was used to assess the severity of liver disease.

### Coronary angiography

The decision to undergo coronary angiography was driven by the clinical discretion of the managing cardiologist, based on the results of stress testing, known CAD, or perceived clinical risk. From coronary angiography reports, percent diameter stenosis values were tabulated according to a standard coronary segmentation model [11]. Severe CAD was defined as ≥50% stenosis in the left main coronary artery or ≥ 70% stenosis in any epicardial coronary artery. Significant CAD was defined as ≥50% stenosis in any epicardial coronary artery [12].

### Outcome assessment

Patients were followed for post-transplantation events of death, cardiac death, myocardial infarction (MI), and surgical or percutaneous coronary revascularization. Events were adjudicated by investigators blinded to clinical data. Outcome status, date of event, and date of last encounter were determined by conducting detailed chart review and Social Security Death Index search. Hospital records and death certificates obtained from the Illinois Department of Public Health were reviewed to determine the cause of death. Cardiac death was defined as death caused by MI, arrhythmias, or heart failure.

The primary outcome was the presence of angiographically severe CAD pre-transplantation. The secondary outcomes were post-transplantation composite adverse events: 1) composite endpoint of cardiac death or MI; 2) composite endpoint of cardiac death, MI, or coronary revascularization.

### Statistical analysis

Logistic regression modeling and receiver operating characteristic (ROC) curves were used to determine the predictive value of the sum of risk factors in determining the presence of severe or significant CAD.

Kaplan-Meier curves and the log-rank test were used to analyze event-free survival after transplantation. Univariate Cox regression models were used to determine the hazard of adverse events based on risk factors, which was expressed as hazard ratio (HR) and 95% confidence interval (CI). The proportional hazards assumption with respect to Cox regression modeling was confirmed using log-minus-log survival plots. SPSS version 23 software package (IBM-Armonk, NY) was used for all statistical analyses. The study was approved by the institutional review board of Rush University Medical Center.

## Results

We identified 220 patients (38.6% female, mean age 55 ± 11 years) who underwent liver transplantation at Rush University Medical Center and were followed for a mean of 4.0 ± 1.3 years. The baseline characteristics of the study cohort are detailed in Table 1. The average number of AHA/ACCF risk factors present prior to liver transplantation was 2.3 ± 1.7 and the frequencies of the total risk factors are listed in Table 1.

**Table 1:** Baseline Characteristics

	All Patients *N* = 220	Angiography Cohort *N* = 42
Age at transplant, years	55 ± 11	60 ± 8
Age > 60	71 (32.3%)	21 (50.0%)
Female sex	85 (38.6%)	17 (40.5%)
Race		
Black	54 (24.5%)	13 (31%)
White	117 (53.2%)	21 (50.0%)
Hispanic	15 (6.8%)	3 (7.1%)
BMI, kg/m^2^	28 ± 8	31 ± 7
Etiology of liver disease		
Hepatitis C	101 (40.6%)	16 (38.1%)
Alcoholic	51 (20.5%)	5 (11.9%)
Non-Alcoholic Steatohepatitis	22 (8.8%)	10 (23.8%)
Autoimmune	22 (8.8%)	2 (4.8%)
Hyperlipidemia	29 (13.2%)	11 (26.2%)
Hypertension	87 (39.5%)	20 (47.6%)
Diabetes Mellitus	74 (33.6%)	19 (45.2%)
Tobacco Use	27 (12.3%)	7 (16.7%)
Congestive Heart Failure	8 (3.6%)	5 (11.9%)
Ejection Fraction	62 ± 8%	64 ± 11
Left ventricular hypertrophy	14 (6.4%)	3 (7.1%)
Cerebrovascular disease (CVA/TIA)	13 (5.9%)	6 (14.3%)
Peripheral Arterial Disease	14 (6.4%)	6 (14.3%)
Known CAD	29 (13.2%)	15 (35.7%)
History of MI	13 (5.9%)	8 (19.0%)
History of PCI	17 (7.7%)	11 (26.2%)
History of CABG	7 (3.2%)	4 (9.5%)
MELD Score, mean ± SD	21 ± 11	21 ± 12
AHA/ACCF risk factors, mean ± SD	2.3 ± 1.7	3.0 ± 1.8
AHA/ACCF risk factors, n		
0–1	77 (35%)	10 (23.8%)
2	54 (24.5%)	11 (26.2%)
3	38 (17.3%)	5 (11.9%)
4	28 (12.7%)	6 (14.3%)
5 or more	23 (10.5%)	10 (23.8%)

Data are presented as mean ± SD or n (%)

*AHA/ACCF* American Heart Association/American College of Cardiology Foundation, *BMI* body mass index, *CABG* coronary artery bypass grafting, *CAD* coronary artery disease, *CVA* cerebrovascular accident, *MELD* model End-Stage Liver Disease, *MI* myocardial infarction, *PCI* percutaneous coronary intervention, *TIA* transient ischemic attack

**Fig. 1:**
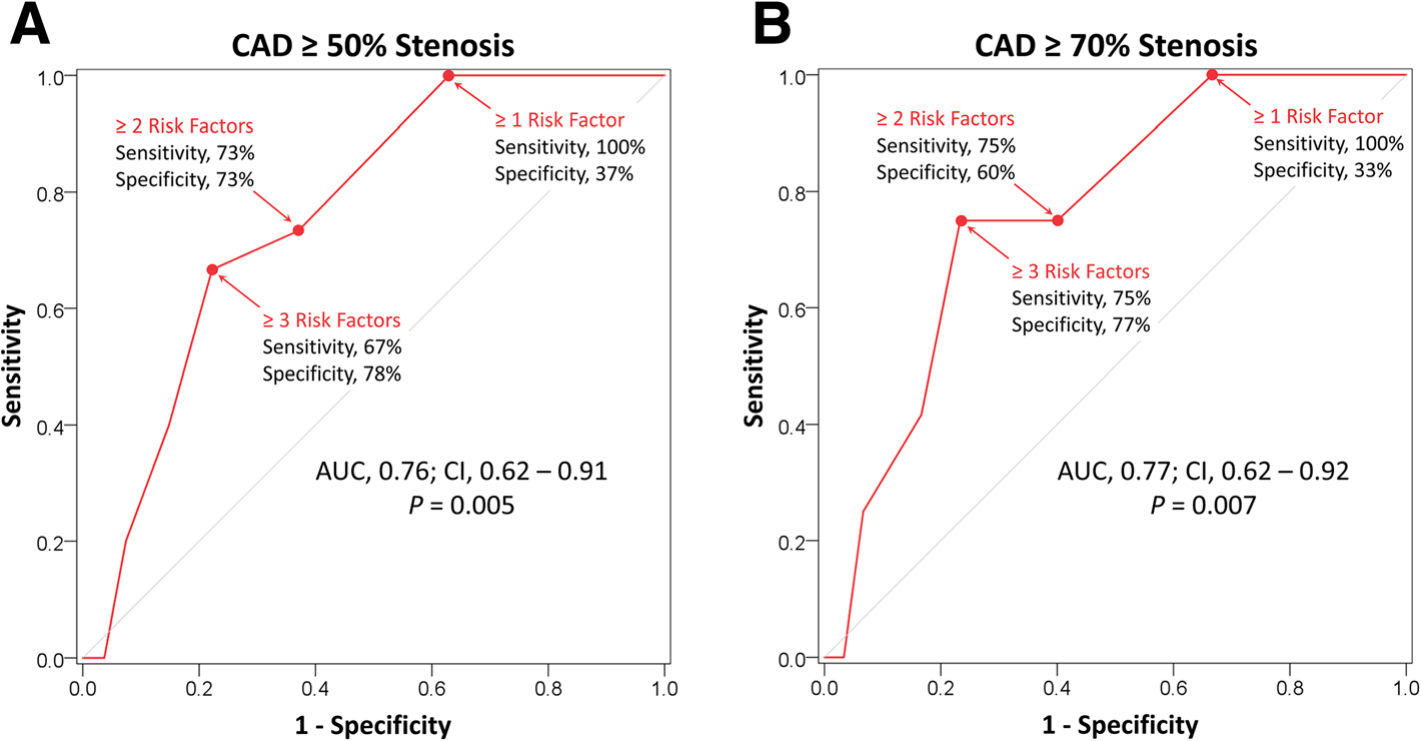
Value of the Sum of AHA/ACCF Risk Factors in Determining the Presence of Coronary Artery Disease. The graphs depict receiver operating characteristic curves of the sum of risk factors as a predictor of significant (**a**) and severe (**b**) coronary artery disease. Significant CAD is defined as ≥50% stenosis in any epicardial coronary artery. Severe CAD is defined as ≥50% stenosis in the left main coronary artery or ≥ 70% stenosis in any epicardial coronary artery. AUC, area under the curve; CAD, coronary artery disease; CI, 95% confidence intervals

Among the 220 liver transplantation recipients, 42 (19%) underwent pre-transplantation coronary angiography. The baseline characteristics of the angiography cohort are outlined in Table 1. Among the 42 patients who underwent an angiogram, 32 had non-invasive testing prior to angiography (26 dobutamine stress echocardiography and 6 pharmacologic stress radionuclide myocardial perfusion imaging). The remaining 10 subjects had established CAD and underwent coronary angiography directly, based on the discretion of the managing cardiologist. Among those who underwent angiography, 15 (35.7%) had significant CAD, while 12 (28.6%) had severe CAD. The ROC analyses demonstrated that the sum of AHA/ACCF risk factors was associated with an area under the curve of 0.76 and 0.77 for detection of significant and severe CAD, respectively (Fig. 1a, b). The cumulative number of risk factors was associated with CAD with an odds ratio of 1.72 per risk factor (CI, 1.12–2.65; *P* = 0.013). A threshold of ≥2 risk factors provided 75% sensitivity and 60% specificity for detection of severe CAD while a threshold of ≥3 risk factors provided similar sensitivity at 75% but superior specificity at 77% for detection of severe CAD (Fig. 1b). Among patients who underwent angiography, 6 subjects had coronary revascularization (5 percutaneous and 1 surgical) prior to liver transplantation.

**Fig. 2:**
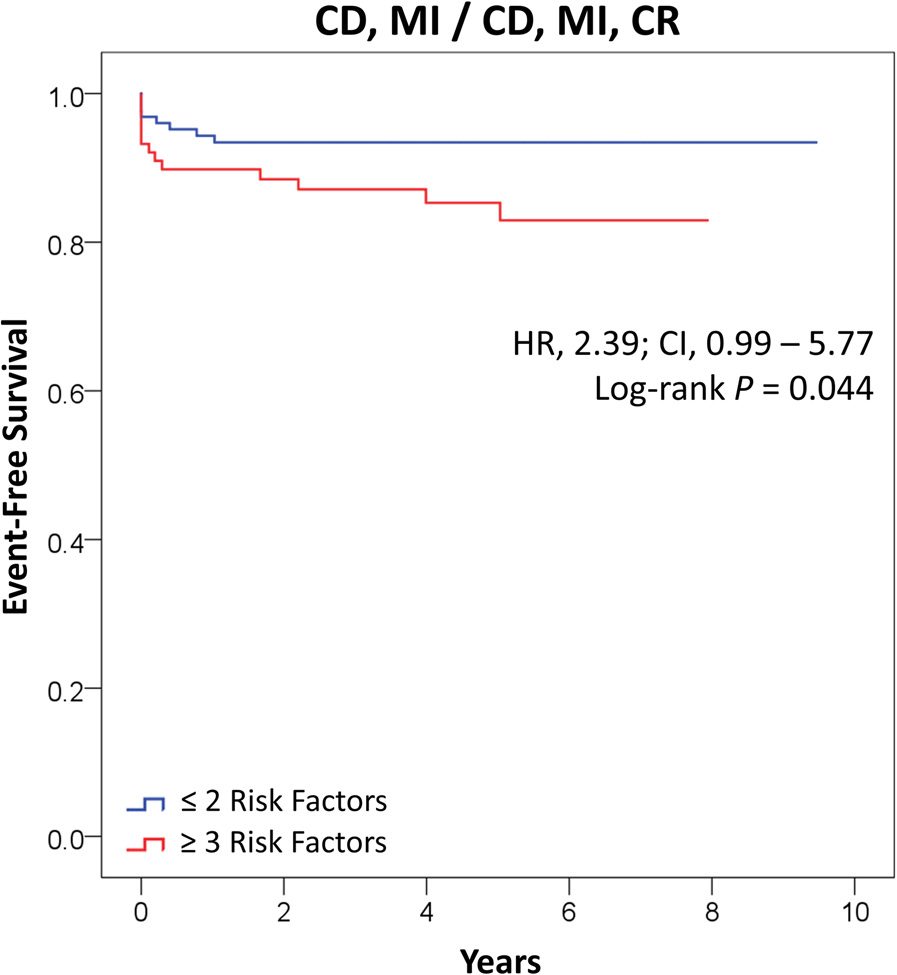
Event-free Survival Based on AHA/ACCF Risk Factors. The graph depicts Kaplan-Meier survival curves for the composite outcome of cardiac death or myocardial infarction and the composite outcome of cardiac death, myocardial infarction, or coronary revascularization. * The secondary outcomes were identical since the 2 coronary revascularization events occurred shortly after MI. CD, cardiac death; MI, myocardial infarction; CR, coronary revascularization

During follow-up, there were a total of 82 deaths of which 17 were cardiac. Major causes of non-cardiac death included malignancy, sepsis, and hemorrhage. Additionally, 5 MIs and 2 surgical coronary revascularizations were observed during post-transplantation follow-up, but there were no percutaneous coronary intervention events. Both revascularization events occurred shortly after MI; thus, there were 21 identical secondary outcomes of composite cardiac death or MI and composite cardiac death, MI or coronary revascularization. As shown in Fig. 2, having ≥3 AHA/ACC risk factors was associated with increased secondary outcomes of cardiac death or MI and cardiac death, MI, or coronary revascularization (HR, 2.39; *P* = 0.044). On the other hand, using a threshold of ≥2 AHA/ACCF risk factors provided insignificant predictive value of event-free survival for both secondary outcomes (*P* values = 0.626).

## Discussion

This is the first study to validate the diagnostic and prognostic utility of risk factors set forth in the 2012 AHA/ACCF Scientific Statement on evaluation of CAD in liver transplantation candidates. Moreover, we demonstrated that the presence of at least 3 risk factors has both high sensitivity and specificity for detecting severe CAD and is associated with increased risk of adverse cardiac events. Therefore, we determined that having 3 or more of these risk factors is an appropriate threshold to justify non-invasive stress testing prior to liver transplantation in patients without cardiac symptoms.

Cardiac risk assessment in asymptomatic patients undergoing liver transplantation is controversial. Historically, patients with end-stage liver disease were assumed to have a lower incidence of CAD compared to the general population, as it was thought that the cirrhotic state confers a protective effect against the development of CAD given decreased hepatic production of cholesterol and peripheral vasodilation with normal to lower blood pressures [7, 13]. In addition, patients with cirrhosis tend to have increased estrogen levels, which are thought to have protective effects from atherosclerosis [13, 14]. Despite these hemodynamic and metabolic effects of cirrhosis, multiple studies have demonstrated that there is a higher than expected prevalence of CAD in liver transplantation candidates [5]. In their study of 100 consecutive liver transplantation candidates, Poulin et al. found that 20% of the cohort had severe CAD [6]. In their review of CAD assessment of liver transplantation candidates, Ehtisham et al. reported that the prevalence of CAD ranged from 2.9 to 28% [7]. Finally, in 101 liver transplantation patients, McAvoy et al. found that 19.8% of patients had a high burden of coronary artery calcification (> 400 Agatston units), a marker of atheromatous plaque [15]. Importantly, CAD has been shown to be a major cause of morbidity and mortality post liver transplantation [1–4, 16]; therefore, cardiac assessment and risk factor modification prior to liver transplantation is essential.

Despite general agreement that identification of coronary disease prior to liver transplantation is valuable, there are considerable differences in approach to cardiac risk assessment when appraising the two major societal guidelines currently available for pre transplantation cardiac workup. In its 2013 practice guideline, the American Association for the Study of Liver Diseases (AASLD) recommended that all liver transplantation candidates undergo non-invasive testing with stress echocardiography as a screening test for CAD, followed by coronary angiography if necessary [17]. The majority of patients with end-stage liver disease are unable to exercise and require pharmacologic stress. Even with maximum doses of dobutamine and atropine, many patients are unable to achieve target heart rate due to baseline autonomic dysfunction. Similarly, as patients with end-stage liver disease are peripherally vasodilated at baseline, the tolerability and effectiveness of vasodilator stress myocardial perfusion imaging has been questioned [5].

In contrast to the AASLD, the AHA/ACCF recommends cardiac risk assessment on the basis of seven risk factors and suggests that non-invasive stress testing may be considered when “multiple” risk factors are present. The committee does not clearly define the number of risk factors to prompt stress testing, and suggested that 3 or more may be reasonable [8]. Not only has the risk assessment model proposed by the AHA/ACCF never been validated, but the uncertainty of these recommendations (class IIB - evidence C) further highlights the need for studies addressing cardiac risk assessment prior to liver transplantation and whether stratification can lead to improved outcomes.^5^ Our study validated the risk assessment approach proposed by the AHA/ACCF Scientific Statement and demonstrated that the presence of at least 3 or more risk factors is predictive of obstructive CAD and post-transplantation cardiac outcomes. Having fewer or no risk factors provides low yield for CAD and predicts favorable cardiac prognosis.

Given the prevalence of CAD in liver transplantation candidates and known poorer outcomes in those with baseline cardiac disease, clinicians can use the sum of AHA/ACCF risk factors as a starting point in evaluating asymptomatic liver transplantation candidates to determine the benefit of further non-invasive testing and prognosticate post-transplantation outcomes.

### Limitations

The retrospective single-center design is an obvious limitation. Moreover, the study did not examine the performance of the AHA/ACCF risk factors in all liver transplantation candidates, but rather only liver recipients. A prospective evaluation of the AHA/ACCF Scientific Statement is needed.

## Conclusion

The clinical risk factors outlined by the 2012 AHA/ACCF Scientific Statement on the cardiac evaluation of liver transplantation candidates provides diagnostic and prognostic utility in identifying patients with severe CAD and at increased risk of cardiac events following liver transplantation. The presence of at least 3 or more of these risk factors seems to be an optimal threshold for initiating non-invasive stress testing to further investigate cardiac risk in asymptomatic liver transplantation candidates.

## Abbreviations

AHA/ACCF: American Heart Association/American College of Cardiology Foundation
AUC: Area under the curve
CAD: Coronary artery disease
CI: Confidence interval
HR: Hazard ratio
MI: Myocardial infarction
ROC: Receiver operating characteristic
